# Goal-directed fluid therapy- a survey of anaesthetists in the UK, USA, Australia and New Zealand

**DOI:** 10.1186/1471-2253-13-5

**Published:** 2013-02-22

**Authors:** Sanket Srinivasa, Arman Kahokehr, Mattias Soop, Matthew Taylor, Andrew G Hill

**Affiliations:** 1 Department of Surgery, South Auckland Clinical School, Middlemore Hospital, University of Auckland, Auckland, New Zealand; 2 Department of Surgery, North Shore Hospital, University of Auckland, Auckland, New Zealand; 3 Department of Anaesthesia, Middlemore Hospital, Auckland, New Zealand

**Keywords:** Intravenous fluid, Goal-directed fluid therapy, Perioperative care, Surgery

## Abstract

**Background:**

Goal-directed fluid therapy (GDFT) has been shown to reduce complications and hospital length of stay following major surgery. However, there has been no assessment regarding its use in clinical practice.

**Methods:**

An electronic survey was administered to randomly selected anaesthetists from the United Kingdom (UK, n = 2000) and the United States of America (USA, n = 2000), and 500 anaesthetists from Australia/New Zealand (AUS/NZ). Preferences, clinical use and attitudes towards GDFT were investigated. Results were collated to examine regional differences.

**Results:**

The response rates from the UK (n = 708) and AUS/NZ (n = 180) were 35%, and 36% respectively. The response rate from the USA was very low (n = 178; 9%). GDFT use was significantly more common in the UK than in AUS/NZ (p < 0.01). The Oesophageal Doppler Monitor was the most preferred instrument in the UK (n = 362; h76%) with no clear preferences in other regions. GDFT was most commonly utilised in major abdominal surgery and for patients with significant comorbidities. The commonest reasons stated for not using GDFT were either lack of availability of monitoring tools (AUS/NZ: 57 (70%); UK: 94 (64%)) or a lack of experience with instruments (AUS/NZ: 43 (53%); UK: 51 (35%)). A subset of respondents (AUS/NZ: 22(27%); UK: 45 (30%)) felt GDFT provided no perceived benefit. Enthusiasm towards the use of GDFT in the absence of existing barriers was high.

**Conclusion:**

Several hypotheses were generated regarding important differences in the use of GDFT between anaesthetists from the UK and AUS/NZ. There is significant interest in utilising GDFT in clinical practice and existing barriers should be addressed.

## Background

Over the last decade, there has been a renewed emphasis on research investigating perioperative fluid therapy [1,2]. This has been complemented by a surge in research exploring optimised perioperative care [3-5]. An increasing body of work has now highlighted the importance of physiologically-guided, individualised fluid administration in keeping with the broad principles recommended by Moore and Shires over 40 years ago [6,7]. This concept is known as goal–directed fluid therapy (GDFT).

GDFT therapy primarily involves the administration of intravenous fluids to optimise pre-defined, patient-specific clinical proxies of tissue perfusion [7]. The individual parameters used to guide therapy are measures of cardiovascular function and vary depending on the monitoring system used [7]. In practice, GDFT involves repeated administration of small boluses of intravenous fluids- often colloids- until a certain target or plateau is reached. A baseline infusion of crystalloids is typically given.

A number of randomised trials and subsequent meta-analyses in various surgical settings have shown that GDFT confers clinical benefits such as decreased postoperative morbidity and decreased hospital length of stay as compared to traditional, liberal fluid therapy [8-14]. This is thought to be on the basis of treating occult hypovolaemia, preventing fluid overload or correcting a preoperative functional intravascular deficit early in the intraoperative phase to decrease the post-surgical inflammatory response [9-11]. The National Institute for Health Research and the Health Technology Assessment committee in the United Kingdom (UK) found in their systematic review that GDFT using the Oesophageal Doppler Monitor (ODM) provides clinical benefits in major surgery and is cost effective [15]. The National Health Service Centre for Evidence-based Purchasing also concluded that the cost of the ODM would be offset by the clinical benefits seen [16]. The use of GDFT tools is also reimbursed in the United States of America (USA) by the Center for Medicare and Medicaid Services who have stated that the use of the Oesophageal Doppler Monitor (ODM) is both “reasonable and necessary” for operative patients requiring fluid optimisation [17,18]. As a result, a number of guidelines from professional groups have recommended the use of GDFT in major surgery in selected cases [19,20].

However, some authors have expressed reservations regarding the proposed benefits of GDFT for all patients. The criticisms include a paucity of trials within an optimised perioperative care environment and no comparison to intraoperative fluid restriction, which is also recognised as beneficial in a similar cohort of patients [2,14,21,22]. A recent qualitative review has also outlined several important unanswered questions in the context of GDFT in colorectal surgery [23]. Moreover, questions persist regarding the best monitoring system, the ideal intraoperative fluid and clinical situations when GDFT is appropriate [7,23,24]. Therefore, there is clinical and academic equipoise regarding the proposed benefits or otherwise of GDFT.

For those in favour of GDFT, the translation of evidence into practice can be a significant hurdle [25,26]. Clinical benefits necessitate proven interventions to be suitably implemented. Selective implementation of interventions can often fail to replicate the benefits observed in trials [25]. It is thus important to assess the current practice and attitudes of clinicians and to identify barriers that may prevent the implementation of evidence-based practice [26,27]. Therefore, we conducted a survey of anaesthetists nationally and internationally to investigate various characteristics regarding GDFT across Australasia, the United Kingdom (UK) and the USA. Specific aims included examining the disparity in clinical uptake of GDFT, situations within which it was used and the preferred tools as well as the barriers to adoption of this technique.

## Methods

Ethical approval was obtained from the Northern X Regional Ethics Committee (NTX/10/EXP/147). An electronic survey was created using a commercially available internet-based service [28]. The survey was administered electronically to 2000 randomly chosen members of the Association of Anaesthetists of Great Britain and Ireland (AAGBI), 2000 randomly chosen members of the American Society of Anesthesiology (ASA) and to 500 randomly chosen members of the Australia and New Zealand College of Anaesthetists (ANZCA) as per the conditions stipulated by each organisation. Randomisation and survey admitration was facilitated by the individual member organisations with respondents remaining anonymous to the investigators. One reminder was sent to the non-responders 4 weeks after the first invitation. The questions from the survey are presented in Table 1. Results were subsequently collated and the two-tailed Fisher’s exact test was used to assess categorical outcomes. A p value of less than 0.05 was considered statistically significant.

**Table 1:** Survey questions

**Questions**	**Options**
Which country do you work in?	
What is your place of practice? (choose as many as applicable)	Tertiary Hospital/District (Community) Hospital/Private practice
What is your subspecialty interest? (up to three choices)	Abdominal Surgery/Non-abdominal general surgery/Orthopaedic surgery/Urology/Cardiothoracic surgery/Obstetrics/Gynaecology/Vascular Surgery/Neurosurgery/Otorhinolaryngology/Ophthalmology/Plastic Surgery/Paediatric Surgery/Trauma/Burns
What is your current position?	Trainee/Consultant < 5 years/Consultant 5–10 years/Consultant > 10 years
**For adult patients having major elective surgery…**	
What is your preferred intraoperative crystalloid fluid?	Normal saline/Balanced salt solution/Dextrose saline/No preference
What is your preferred intraoperative colloid fluid?	Succinylated gelatin/Tetrastarch/Pentastarch/Hetastarch/Albumin/Nil/No preference/Other
What is your preferred intraoperative pressor? (please state type)	
For adult patients undergoing major elective surgery, do you use goal-directed fluid therapy?	Always/Sometimes/Never
If you use goal directed fluid therapy sometimes, when? (Choose as many as applicable)	Major surgery/Patients with significant comorbidities/Particular operations (state type)/Depending on Instrument availability/Random/Other
If yes, what kind of surgery do you use goal directed fluid therapy in? (choose as many as applicable)	Abdominal Surgery/Non-abdominal general surgery/Orthopaedic surgery/Urology/Cardiothoracic surgery/Obstetrics/Gynaecology/Vascular Surgery/Neurosurgery/Otorhinolaryngology/Ophthalmology/Plastic Surgery/Paediatric Surgery/Trauma/Burns
What are your preferred tools to conduct Goal Directed Fluid Therapy? (choose up to 3)	ODM/PPV monitors/SVV monitors/LiDCO/PAC/SvO2/PVI
If you never use goal directed fluid therapy, why? (choose as many as applicable)	Lack of resources/Nil advantage perceived/Too labour intensive/Unsuitable patients/Lack of experience with instruments
If existing barriers were removed (e.g. lack of resources/training), would you like to use Goal Directed Fluid Therapy?	Yes/No/Undecided

*ODM*: Oesophageal Doppler monitor; *PPV*: Pulse pressure variation; *SVV*: Stroke volume variation; *LiDCO*: Lithium dilution coefficient; *PAC*: Pulmonary artery catheter; *SvO2*: Venous oxygen saturations; *PVI*: Pleth variability index.

## Results

The demographic characteristics of respondents and the response rates are presented in Table 2. The response rates in the UK and AUS/NZ were 35% and 36% respectively. The response rate in the USA was 9%, thereby limiting the validity of any deductions from these data. Amongst responders, the survey completion rate was high across all three regions (UK: 94%; AUS/NZ: 89%). The two commonest subspecialty interests were orthopaedic surgery (AUS/NZ: 76 (46%); UK: 240 (37%)) and abdominal surgery (AUS/NZ: 71(43%); UK: 300 (46%)). Intraoperative fluid preferences are as per Table 3. The two most favoured pressors in the UK and AUS/NZ were metaraminol (UK: 281 (42%); AUS/NZ: 102 (59%)) and phenylephrine (UK: 142 (22%); AUS/NZ: 36 (21%)).

**Table 2:** Respondent characteristics

	**AUS/NZ**	**UK**	p-value*
**No of responders (%)**	180	708	
**Response rate**	36%	35%	
**Place of practice (%):**			
**Tertiary Hospital District Hospital**	122 (69)	360 (51)	<0.01
**Private Practice**	34 (19)	377 (54)	<0.01
	68 (38)	89 (13)	<0.01
**Experience (%):**			
**Trainee**	2 (1)	168 (24)	<0.01
**Consultant <5 years**	27 (15)	161 (23)	0.02
**Consultant 6–10 years**	28 (16)	98 (14)	0.63
**Consultant >10 years**			
	122 (68)	269 (39)	<0.01

*Two-tailed Fisher’s exact test.

**Table 3:** Intraoperative fluid preferences

	**AUS/NZ**	**UK**
Crystalloid (%)	n = 174	n = 692
0.9% Saline	10 (6)	14 (2)
Balanced Salt Solution	162 (93)	662 (96)
Colloid (%)		
Succinylated gelatine	43 (24)	292 (42)
Tetrastarch	30 (17)	98 (14)
Pentastarch	29 (16)	50 (7)
Hetastarch	29 (16)	124 (18)
Albumin	14 (8)	1
No preference	26 (15)	98 (14)
Other	8 (4)	29 (4)

**Figure 1:**
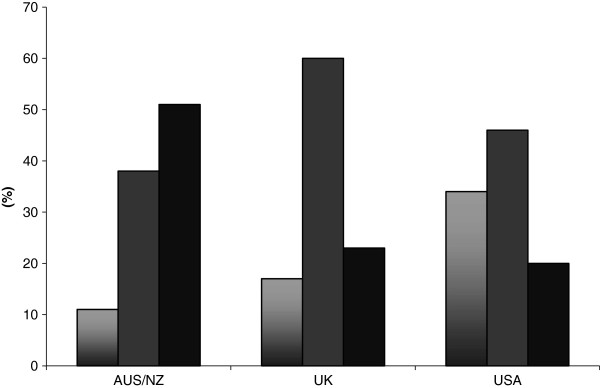
**Do you use Goal-directed fluid therapy? (Columns from left to right respectively: Always; Sometimes; Never).** Always/Sometimes: USA vs. UK, p = 0.36; USA vs. NZ, p < 0.01; UK vs. NZ, p < 0.01.

The use of GDFT is shown in Figure 1. GDFT use was significantly more common in the UK as compared to AUS/NZ (p < 0.01). In AUS/NZ, GDFT was most commonly utilised in abdominal surgery (n = 60; 80%), followed by orthopaedic surgery (n = 27; 36%). In the UK, GDFT was most commonly utilised in abdominal surgery (UK: n = 428 (89%)), followed by vascular surgery (UK: n = 146 (30%)).

**Figure 2:**
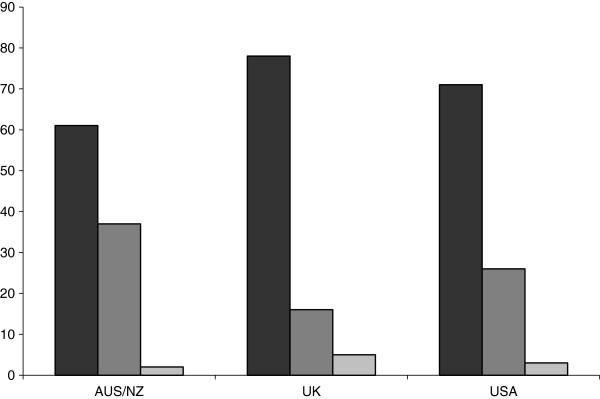
Would you like to use Goal-Directed Fluid Therapy? (Columns from left to right respectively: Yes/No/Undecided).

The situations when GDFT is utilised and preferred tools to conduct GDFT are shown in Tables 4 and 5 respectively. The commonest reasons stated for not using GDFT were either lack of availability of monitoring tools (AUS/NZ: 57 (70%); UK: 94 (64%)) or a lack of experience with instruments (AUS/NZ:43 (53%); UK: 51 (35%)). A subset of respondents cited “nil advantage perceived” as the reason for not using GDFT (AUS/NZ: 22(27%); UK: 45 (30%)). Enthusiasm towards GDFT in the absence of existing barriers (e.g. lack of equipment or experience) is shown in Figure 2.

**Table 4:** Situations when goal-directed fluid therapy is used

**Indication (%)**	**AUS/NZ (n = 87)**	**UK (n = 523)**
Major Surgery	50 (69)	326 (73)
Significant Comorbidities	51 (70)	367 (83)
Specific Operations	20 (27)	107 (24)
Instrument availability	19 (26)	125 (28)
Random	0	11 (3)

**Table 5:** Preferred tools for goal-directed fluid therapy

**Tools (%)**	**AUS/NZ (n = 78)**	**UK (n = 519)**
ODM	11 (19)	362 (76)
PPV	26 (45)	97 (20)
SVV	21 (36)	71 (15)
LiDCO	1 (2)	93 (20)
PAC	17 (29)	35 (7)
SvO2	12 (21)	88 (19)
PVI	3 (5)	8 (2)

*ODM*: Oesophageal doppler monitor; *PPV*: Pulse pressure variation; *SVV*: Stroke volume variation; *LiDCO*: Lithium dilution coefficient; *PAC*: Pulmonary artery catheter; *SvO2*: Venous oxygen saturation; *PVI*: Pleth variability index.

The respondents from the USA indicated a preference towards GDFT in abdominal surgery (n = 97; 80%) with similar barriers to GDFT as described in other regions such as lack of availability of tools (n = 19; 59%), lack of experience (n = 13; 40%) and nil perceived advantage (n = 13; 40%). The most favoured pressor in the USA was phenylephrine (68; 40%), followed by ephedrine (41; 24%).

## Discussion

This survey of 1067 anaesthetists from the UK, AUS/NZ and the USA reveals important differences in practice with regards to intraoperative fluid therapy and GDFT specifically. The response rates were moderate or low, especially in the USA, and it is likely that the present data represents views of a self-selected group among the anaesthetists who were randomly invited to participate. Such selection could be based on strong positive or negative views on GDFT. Nevertheless, certain observations can be made and hypotheses generated regarding the interest in and barriers to GDFT in the UK and AUS/NZ. The poor response rate from the USA limits the validity of any statements regarding practice in this region except to speculate that there is comparatively lesser interest in this topic.

The use of GDFT seems to be significantly less prevalent in AUS/NZ compared to the UK amongst respondents in this survey. The majority of the respondents were involved in major abdominal surgery and orthopaedic surgery and used GDFT in patients with significant comorbidities. The ODM is the most commonly utilised instrument in the UK with significant variation in preferences in other regions. The most significant barriers to conducting GDFT were either a lack of availability of monitoring tools or a lack of experience with instruments. Even though a proportion of respondents from all regions remain unconvinced of the benefits of GDFT, there was significant enthusiasm overall towards trialling GDFT if barriers were to be removed.

The use of GDFT was most common in the UK which may have been made possible by governmental endorsement and funding of the ODM specifically with demonstrated cost-effectiveness [15,16]. Nationally-driven implementation may also potentially overcome the problems associated with silo budgeting as GDFT represents an anaesthetic intervention- thereby adding cost to clinical departments of anaesthesia- to provide financial benefits for surgical services such as decreased hospital length of stay. This represents a potential blueprint for other regions to follow. For clinicians, the recently published GIFTASUP guidelines which recommend mandatory use of GDFT in major abdominal surgery may have also provided further impetus [20].

Moreover, a significant portion of the evidence supporting GDFT originates from the UK [9,11,29]. The recent guidelines from the European Enhanced Recovery After Surgery (ERAS) group have also shown enthusiasm towards GDFT and the ODM and, as ERAS protocols are instituted across the UK, GDFT has been integrated into practice as well [19,30,31]. This has likely led to the emergence of the ODM as the preferred tool to conduct GDFT in the UK. In contrast, the principles of optimised perioperative care have shown reduced penetrance in AUS/NZ with persisting scepticism regarding benefits [32,33]. In AUS/NZ, there appears to be no clear preference with regards to tools for GDFT.

The barriers identified to the use of GDFT appear eminently solvable especially if the observed benefits from trials are replicated in clinical practice [13]. Many of the instruments used to conduct GDFT are simple to use and the learning curve for the ODM can be overcome after 12 insertions [34]. However, it should be noted that in the context of abdominal surgery, restrictive fluid regimens have also shown similar benefits to GDFT and the majority of the trials investigating GDFT have not been conducted in an environment of standardised, optimised perioperative care [14,23].

A proportion of people from all the regions surveyed remain sceptical regarding the proposed benefits of GDFT. To an extent, this is justified as important questions remain unanswered, such as efficacy in settings where fluid restriction has been shown to be beneficial [21-23]. Nonetheless, it is interesting to note that in the absence of barriers, a high proportion of respondents would be willing to consider GDFT into their practice. This suggests that future randomised trials or selective clinical implementation of GDFT remain feasible and are required.

Whilst the use of intraoperative crystalloids was largely homogeneous between the regions, there were interesting differences in practice between the three regions with regards to colloid use. There are considerable differences between individual classes of colloids as well as between the individual colloid products themselves [35,36]. Succinylated gelatin solutions were favoured in the UK and AUS/NZ with the latter showing a wide variation in practice. This may be a reflection of the variable availability and cost of these solutions.

There are important limitations to this study which need to be considered alongside the results. The survey was designed on the basis of questions of clinical importance but was not developed with a focus group nor with redundant questions to validate its methodology. Moreover, since the survey aimed towards an overview of GDFT, specifics could not be elucidated regarding any one aspect (e.g. specific barriers). Any barriers identified were the opinion of the respondent and may not be reflective of the institution or the region in its entirety. The number of individuals to be surveyed was limited by the respective professional associations. The colleges facilitated selection of individuals with no author input allowed regarding this. The only selection criterion was to prevent administration of the survey to individuals who had previously replied to other college-led surveys to minimise responder fatigue. In the context of a low-moderate response rate, it cannot be assumed with certainty that it is representative of the anaesthetic community in its entirety.

## Conclusion

There are important differences in fluid administration and the use of GDFT between anaesthetists from the UK and AUS/NZ. The identified barriers can potentially be overcome and though some clinicians remain unconvinced, there is significant interest in utilising GDFT in clinical practice. Future directions from this work can include selective implementation of GDFT or further clinical studies.

## Competing interests

The authors are currently running a randomised trial exploring Oesophageal Doppler-guided fluid therapy in colonic resections within an enhanced recovery after surgery protocol. (NCT00911391). The Oesophageal Doppler machine for this trial has been lent by Pharmaco New Zealand. All probes have been bought at regular cost and Pharmaco New Zealand has had no input into the design or conduct of the trial and will not have any influence on publication. The authors of this manuscript run an annual course on the principles of optimised perioperative care (http://webstarts.com/aerascourse) and have received sponsorship from Pharmaco NZ for this event. Pharmaco NZ has no input into the course content or delivery.

## Authors’ contributions

SS- designed the survey, analysed the data, wrote the manuscript. AK- analysed the data, edited the manuscript. MS- edited the survey, revised the manuscript. MT- conceived the idea for the project, revised the manuscript. AH- conceived the idea for the project, designed the survey, assisted in data analysis and revised the manuscript. All authors read and approved the final manuscript.

## Pre-publication history

The pre-publication history for this paper can be accessed here:

http://www.biomedcentral.com/1471-2253/13/5/prepub
